# The significance of preclinical anti-BP180 autoantibodies

**DOI:** 10.3389/fimmu.2022.963401

**Published:** 2022-08-08

**Authors:** Yosuke Mai, Kentaro Izumi, Shoko Mai, Hideyuki Ujiie

**Affiliations:** Department of Dermatology, Faculty of Medicine and Graduate School of Medicine, Hokkaido University, Sapporo, Japan

**Keywords:** bullous pemphigoid, autoantibodies, BP180, anti-BP180 autoantibodies, autoimmune disease, aging, diabetes mellitus, neurological disease

## Abstract

Bullous pemphigoid (BP) is the most common autoimmune subepidermal blistering disease. Although the pathomechanism of BP onset has yet to be elucidated in detail, BP autoantibodies targeting two hemidesmosomal components, BP180 and BP230, are known to play a pivotal role in BP pathogenesis. Thus, the detection and measurement of BP autoantibodies are necessary for diagnosing BP and monitoring the disease activity. Immune assays such as immunofluorescence microscopy, immunoblotting, and ELISAs using BP180 and BP230 detect BP autoantibodies in most BP cases with high specificity; however, BP autoantibodies are sometimes detected in BP patients before the onset of this disease. BP autoantibodies that are detected in patients without typical tense blisters are defined as “preclinical BP autoantibodies”. These preclinical BP autoantibodies are detected even in a low percentage of normal healthy individuals. Although the importance of preclinical BP autoantibodies remains elusive, these autoantibodies might be a potential risk factor for subsequent BP development. Therefore, previous comparative epidemiological studies have focused on the prevalence of preclinical BP autoantibodies in populations susceptible to BP (e.g., the elderly) or in diseases with a higher risk of comorbid BP. This mini-review summarizes the literature on the prevalence of preclinical BP autoantibodies in patients with various conditions and diseases, and we discuss the significance of preclinical BP autoantibody detection.

## Introduction

Bullous pemphigoid (BP), a major autoimmune blistering disease, is characterized clinically by tense blisters and/or urticarial erythema and histologically by subepidermal blisters with abundant eosinophilic infiltration (1, 2). Autoantibodies directed to the basement membrane zone (BMZ) play an essential role in BP pathogenesis. BP autoantibodies mainly target two hemidesmosomal components: BP180 and BP230, and anti-BP180 autoantibodies have been recognized as the major pathogenic autoantibodies in BP (3–5). Furthermore, the 16^th^ non-collagenous (NC16A) domain has been identified as a major pathogenic epitope of BP autoantibodies because 80–90% of BP sera show positive reactivity to the NC16A domain within BP180 (6, 7).

Regarding BP diagnosis, the detection of BP autoantibodies is necessary in addition to clinical and histological findings (1, 8). However, the presence of BP autoantibodies is not a sufficient condition for diagnosis. In fact, the production of BP autoantibodies precedes clinical tense blister formation in certain BP cases (9). Therefore, previous studies have investigated the prevalence of preclinical BP autoantibodies as a potential risk for subsequent BP development in various conditions, including aging, pruritus, collagen diseases, and neurological disorders.

### Detection methods for BP autoantibodies

BP autoantibodies are present in BP patients in two forms: tissue-bound autoantibodies in lesional skin and circulating autoantibodies in peripheral blood. The former is detected with direct immunofluorescence (DIF) microscopy; the latter is detected with indirect immunofluorescence (IIF) microscopy, ELISA, and immunoblotting. With DIF, which is the most sensitive method of finding BP autoantibodies, those antibodies show up as the linear deposition of IgG and/or C3 at the dermal-epidermal junction of lesional skin (10). Several studies suggested a new subset of IgG4-dominant BP, which predominantly has IgG4 autoantibodies without complement activation at the BMZ (11–13). However, the linear deposition of C3 at the BMZ is a promising diagnostic sign for BP (14). The limitation of DIF is that it is difficult to differentiate autoantibodies of BP from those of other subepidermal autoimmune blistering diseases.

IIF semi-quantitates the titration of circulating BP autoantibodies using skin or esophagus cryosections from a healthy individual. Using 1 M NaCl-split skin sections, IIF allows differentiation between BP and other autoimmune subepidermal blistering skin diseases. In 1 M NaCl-split skin IIF (ssIIF), reactivity against the epidermal side corresponds to BP, while reactivity against the dermal side corresponds to anti-laminin 332 mucous membrane pemphigoid, epidermolysis bullosa acquisita, and anti-p200 pemphigoid (also called anti-laminin gamma 1 pemphigoid). Immunoblotting using epidermal extracts or recombinant proteins of BP autoantigens is also available to detect circulating BP autoantibodies in limited facilities.

ELISA using the BP180 NC16A recombinant protein (BP180 NC16A ELISA) is a commercially available method of detecting and quantifying BP autoantibodies. BP180 NC16A ELISA has a sensitivity of 84.4% and a specificity of 98.9% in diagnosing BP (6). Furthermore, the titration of BP autoantibodies quantified by BP180 NC16A ELISA closely correlates with BP activity (15); thus, BP180 NC16A ELISA is also useful for monitoring BP. Also, an ELISA using tetrameric NC16A recombinant proteins is commercially available (16). A few laboratories have developed ELISAs that use non-NC16A regions of BP180 and full-length BP180 to diagnose BP. Hofmann et al. showed that 54/116 (46.6%) of BP sera reacted to BP180 COOH-terminal regions, i.e., aa1351*–*1497 (17). Using a random BP180 epitope library displayed on a bacteriophage, 31/57 (54.4%) of BP sera reacted to at least one additional region other than BP180 NC16A, aa490*–*562, within the extracellular (36.8%) or intracellular (28.1%) domains of BP180 (18). An ELISA using BP180 aa1080*–*1107 and aa1331*–*1404 detected anti-BP180 autoantibodies in 32/78 (41.0%) of BP sera (19). A multicenter prospective study showed that distinct epitopes of the BP180 ectodomain other than BP180 NC16A are recognized by 95.9% of BP sera (20). An ELISA using human full-length BP180 recombinant proteins (a full-length BP180 ELISA) was able to detect anti-BP180 autoantibodies targeting the NC16A domain as well as other regions of BP180 for 83.5% of BP sera (21).

BIOCHIP is a unique IIF-based serologic diagnostic assay for detecting autoantibodies of autoimmune blistering diseases (22). The BIOCHIP technique provides autoimmune blistering-related antibody profiles of patient sera in a single incubation on the mosaic slide. BIOCHIP contains a recombinant BP180 NC16A protein, and the sensitivity of anti-BP180 NC16A autoantibodies in BP detected by BIOCHIP is 55.3–100.0% (22–31).

Although IIF, WB, ELISA, and BIOCHIP can detect anti-BP180 antibodies, the results can differ. Among BP180 ELISA-positive BP patients, positive rates for IIF, immunoblot, and BIOCHIP were 77.3–83.3%, 69.0–95.5%, and 83.3–100.0%, respectively (25, 26, 29, 32, 33). Low-titer anti-BP180 autoantibodies detected by ELISA may be confirmed by IIF or immunoblotting.

## Prevalence of preclinical anti-BP180 autoantibodies in normal healthy individuals

We reviewed the studies evaluating preclinical anti-BP180 autoantibodies in normal healthy individuals and listed representative studies whose populations exceeded 50 (**Table 1**). BP180 NC16A ELISA detected anti-BP180 autoantibodies in 0.0–4.2% of normal healthy individuals (6, 16, 19, 35–40, 42, 43). Wieland et al. showed that 14 (4.15%) of 337 unaffected subject sera were positive by BP180 NC16A ELISA, and the mean titer of ELISA-positive sera was 16.2 ± 6.3 (mean ± standard deviation, SD, cutoff value <9.0) (39). According to other studies, the mean titers of normal subjects by BP180 NC16A ELISA were 2.1–2.5 (35, 37, 43). The mean titer of ELISA-positive sera was higher in the Wieland et al. study than in the other studies. However, these ELISA-positive sera had no positive reactivities toward a monkey esophagus by IIF (39). ELISAs using the mid-portion (aa490–811) or the C-terminal domain (aa1351–1497) of BP180 detected anti-BP180 autoantibodies in 1.4% and 4.2% of sera from normal healthy individuals, respectively (17).

**Table 1:** The prevalences of anti-BP180 autoantibodies in normal healthy individuals.

Paper	Year	Subjects	Positive rate of anti-BP180 autoantibodies	Subject attributes	Assay
Nakatani et al. (34)	1998	50	0.0%	Normal control sera	Immunoblotting using BP180 NC16A
		50	0.0%	Normal control sera	Immunoblotting using the BP180 C-terminus
Kobayashi et al. (6)	2002	336	1.5%	Normal individuals	BP180 NC16A ELISA
Hofmann et al. (17)	2002	72	1.4%	Normal human sera	ELISA using recombinant BP180 aa490–811
		72	4.2%	Normal human sera	ELISA using recombinant BP180 aa1351–1497
Mariotti et al. (19)	2004	85	1.2%	Healthy volunteers	ELISA using recombinant BP180 aa1080–1107 and 1311–1404
		70	0.0%	Healthy volunteers	BP180 NC16A ELISA
Sakuma-Oyama et al. (35)	2004	60	3.3%	Normal volunteers	BP180 NC16A ELISA
Yoshida et al. (36)	2005	336	1.5%	Normal control sera	BP180 NC16A ELISA
Powell et al. (37)	2005	166	0.0%	Control subjects	BP180 NC16A ELISA
Sitaru et al. (16)	2007	494	2.0%	Healthy donors	BP180 tetrameric NC16A ELISA
Desai et al. (38)	2008	61	31.1%	Healthy subjects and volunteers	Immunoblotting using epidermal extracts
		20	0.0%	Immunoblot-positive subjects	BP180 NC16A ELISA
Wieland et al. (39)	2010	337	4.2%	Unaffected subjects	BP180 NC16A ELISA
van Beek et al. (22)	2012	100	1.0%	Healthy blood donors	BIOCHIP
van Beek et al. (40)	2014	50	0.0%	Blood donors	BP180 NC16A ELISA
		50	0.0%	Blood donors	BP180 tetrameric NC16A ELISA
Prüßmann et al. (41)	2015	7063	0.5%	Normal blood donors	BIOCHIP
Recke et al. (42)	2016	75	2.7%	Healthy controls	BIOCHIP and BP180 NC16A ELISA
Tuusa et al. (43)	2019	140	1.4%	Healthy control subjects	BP180 NC16A ELISA
Mai et al. (44)	2022	1035	2.2%	Normal blood donors	Full-length BP180 ELISA

Regarding immunoblotting, none of the normal control sera showed positive reactivity against GST-fusion proteins containing 42 amino acids of the NC16A region nor 49 amino acids of the C-terminal region of BP180 (34). Immunoblotting using epidermal extracts detected BP autoantibodies in 31% of healthy individuals, while BP180 NC16A ELISA did not detect BP autoantibodies in a population of those who were seropositive to epidermal extracts (38). For BIOCHIP, 0.5–1.0% of healthy blood donors showed reactivity to the NC16A domain of BP180 (22, 41).

An observational study to evaluate the prevalence of anti-BP180 antibodies in healthy volunteers by using a full-length BP180 ELISA was conducted in Japan (44). 2.2% of serum samples from the healthy volunteers possessed anti-BP180 autoantibodies without the volunteers having any skin lesions. The mean titer ± SD of ELISA-positive sera was 13.9 ± 16.4 (cutoff value <4.64), whereas the mean titer of the parent population was 0.8 ± 3.2. In addition, 12/23 (52.1%) of ELISA-positive sera were positive by IIF using monkey esophagus. Intriguingly, the existence of anti-BP180 autoantibodies is associated with a history of bone fracture and the administration of anti-osteoporosis drugs (44).

We also compared the prevalence of preclinical anti-BP180 autoantibodies among the detection systems. The mean ± SD (range) prevalence for immunoblot, ELISA, and BIOCHIP was 10.4 ± 18.0 (0–31.1), 1.6 ± 1.4 (0.0–4.2), 1.4 ± 1.2 (0.5–2.7), respectively (6, 16, 17, 19, 22, 34–44). Although the high prevalence of anti-BP180 autoantibodies detected by immunoblotting in the Desai et al. study was not confirmed by ELISA (38), immunoblotting might detect preclinical anti-BP180 autoantibodies more efficiently than the other detection methods do.

## Prevalence of preclinical anti-BP180 autoantibodies in BP-susceptible populations or in patients with diseases associated with comorbid BP

We also reviewed the studies evaluating preclinical anti-BP180 autoantibodies in various medical conditions and listed the representative studies whose populations exceeded 20 (**Table 2**).

**Table 2:** The prevalences of anti-BP180 autoantibodies in patients with various medical conditions.

Condition	Paper	Year	Subjects	Positive rates of anti-BP180 autoantibodies	Subject attributes	Assay
**Aging or pruritus**						
	Reickhoff-Catoni et al. (45)	1992	24	0.0%*	Control subjects	Immunoblotting using keratinocyte extracts including BP180 and BP230
			83	15.7%*	Patients with pruritic disorders	Immunoblotting using keratinocyte extracts including BP180 and BP230
	Hachisuka et al. (46)	1996	32	0.0%	Normal elderly individuals	Immunoblotting using human or guinea pig epidermal extracts
	Hofmann et al. (47)	2003	25	12%	Elderly patients with pruritic disorders	BP180 NC16A ELISA
	Feliciani et al. (48)	2009	25	0.0%	Elderly patients with immediate-type allergy	ELISA using BP180 aa490–812
			15	13.3%	Elderly patients with pruritic disorders	ELISA using BP180 aa490–812
			25	4.0%	Elderly patients with immediate-type allergy	ELISA using BP180 aa1048–1465
			15	13.3%	Elderly patients with pruritic disorders	ELISA using BP180 aa1048–1465
	Fania et al. (49)	2012	24	4.2%	Elderly patients with allergic disorders	ELISAs using BP180 aa490–812 and aa1048–1465
			21	23.8%	Elderly patients with pruritic dermatoses	ELISAs using BP180 aa490–812 and aa1048–1465
	van Beek et al. (40)	2014	50	0.0%	Blood donors	BP180 NC16A ELISA
			93	0.0%	Elderly patients with non-inflammatory skin diseases	BP180 NC16A ELISA
			78	1.3%	Elderly patients with chronic pruritic skin disorders	BP180 NC16A ELISA
			50	0.0%	Blood donors	BP180 tetrameric NC16A ELISA
			93	3.2%	Elderly patients with non-inflammatory skin diseases	BP180 tetrameric NC16A ELISA
			78	0.0%	Elderly patients with chronic pruritic skin disorders	BP180 tetrameric NC16A ELISA
**Collagen disease**s						
	Kobayashi et al. (6)	2002	336	1.5%	Normal individuals	BP180 NC16A ELISA
			91	1.1%	Collagen disease patients	BP180 NC16A ELISA
	Sitaru et al. (16)	2007	494	2.0%	Healthy donors	BP180 tetrameric NC16A ELISA
			50	4.0%	Systemic scleroderma	BP180 tetrameric NC16A ELISA
			72	1.4%	Systemic lupus erythematosus	BP180 tetrameric NC16A ELISA
			107	1.9%	Rheumatoid arthritis	BP180 tetrameric NC16A ELISA
			50	8.0%	Systemic scleroderma	BP180 NC16A ELISA
			72	4.2%	Systemic lupus erythematosus	BP180 NC16A ELISA
			107	2.0%	Rheumatoid arthritis	BP180 NC16A ELISA
**Neurological diseases**						
	Messingham et al. (50)	2016	23	0.0%	Control subjects	BP180 NC16A ELISA
			24	0.0%	Parkinson’s disease	BP180 NC16A ELISA
			26	3.8%	Dementia	BP180 NC16A ELISA
			23	0.0%	Control subjects	Immunoblotting using the BP180 ectodomain
			24	29.2%	Parkinson’s disease	Immunoblotting using the BP180 ectodomain
			26	3.8%	Dementia	Immunoblotting using the BP180 ectodomain
			23	0.0%	Control subjects	Immunoblotting using the BP180 intracellular domain
			24	8.3%	Parkinson’s disease	Immunoblotting using the BP180 intracellular domain
			26	19.2%	Patients with dementia	Immunoblotting using the BP180 intracellular domain
	Recke et al. (42)	2016	75	2.7%	Healthy controls	BP180 NC16A ELISA
			65	1.5%	Non-inflammatory skin diseases	BP180 NC16A ELISA
			50	4.0%	Parkinson’s disease	BP180 NC16A ELISA
			50	0.0%	Multiple sclerosis	BP180 NC16A ELISA
			75	0.0%	Parkinson’s disease	BP180 NC16A ELISA
			75	6.7%	Other neurological diseases	BP180 NC16A ELISA
			65	1.5%	Healthy controls	BP180 NC16A ELISA
	Kokkonen et al. (51)	2017	38	2.6%	Healthy control subjects	BP180 NC16A ELISA
			111	18.0%	Alzheimer’s disease	BP180 NC16A ELISA
	Tuusa et al. (43)	2019	140	1.4%	Healthy control subjects	BP180 NC16A ELISA
			143	7.7%	Multiple sclerosis	BP180 NC16A ELISA
			35	11.4%	Multiple sclerosis	Full-length BP180 ELISA
			111	6.3%	Alzheimer’s disease	Full-length BP180 ELISA
			35	0.0%	Multiple sclerosis	Immunoblotting using BP180 NC16A
	Wang et al. (52)	2019	100	5.0%	Healthy control subjects	BP180 NC16A ELISA
			100	14.0%	Stroke	BP180 NC16A ELISA
	Wang et al. (53)	2020	50	8.0%	Healthy control subjects	BP180 NC16A ELISA
			48	47.9%	Alzheimer’s disease	BP180 NC16A ELISA
**Diabetes mellitus**						
	Jedlickova et al. (54)	2008	20	10.0%	Non-diabetic patients without pruritus	ELISA using recombinant BP180
			31	9.7%	Non-diabetic patients with pruritus	ELISA using recombinant BP180
			18	0.0%	Diabetic patients without pruritus	ELISA using recombinant BP180
			21	9.5%	Diabetic patients with pruritus	ELISA using recombinant BP180
	Izumi et al. (55)	2019	54	0.0%	Diabetic patients without DPP4i	BP180 NC16A ELISA
			221	1.8%	Diabetic patients with DPP4i	BP180 NC16A ELISA
			54	5.6%	Diabetic patients without DPP4i	Full-length BP180 ELISA
			221	10.9%	Diabetic patients with DPP4i	Full-length BP180 ELISA
**Other skin diseases**						
	Powell et al. (37)	2005	166	3.0%	Control subjects	BP180 NC16A ELISA
			164	4.9%	PUPPP	BP180 NC16A ELISA
	Feliciani et al. (48)	2009	25	0.0%	Elderly patients with immediate-type allergy	ELISA using BP180 aa490–812
			15	13.3%	Elderly patients with pruritic disorders	ELISA using BP180 aa490–812
			25	4.0%	Elderly patients with immediate-type allergy	ELISA using BP180 aa1048–1465
			15	13.3%	Elderly patients with pruritic disorders	ELISA using BP180 aa1048–1465
	Tampoia et al. (24)	2012	40	0.0%	Healthy subjects	BIOCHIP
			54	0.0%	Psoriasis, discoid lupus erythematosus, lichen ruber planus	BIOCHIP
	van Beek et al. (22)	2012	100	1.0%	Healthy blood donors	BIOCHIP
			97	7.2%	Non-inflammatory skin diseases	BIOCHIP
	Fania et al. (49)	2012	24	4.2%	Elderly patients with allergic disorders	ELISAs using BP180 aa490–812 and aa1048–1465
			21	23.8%	Elderly patients with pruritic dermatoses	ELISAs using BP180 aa490–812 and aa1048–1465
	van Beek et al. (40)	2014	50	0.0%	Blood donors	BP180 NC16A ELISA
			93	0.0%	Non-inflammatory skin diseases	BP180 NC16A ELISA
			78	1.3%	Chronic pruritic skin disorders	BP180 NC16A ELISA
			50	0.0%	Blood donors	BP180 tetrameric NC16A ELISA
			93	3.2%	Non-inflammatory skin diseases	BP180 tetrameric NC16A ELISA
			78	0.0%	Chronic pruritic skin disorders	BP180 tetrameric NC16A ELISA

*These data include autoantibodies targeting BP180 and BP230.

PUPPP, pruritic urticarial papules and plaques of pregnancy.

### Aging and pruritus

BP predominantly occurs in individuals over age 60 (56). In a retrospective observational study of 869 patients with BP in the United Kingdom, the median age at presentation for BP was 80 years (57). Two epidemiological studies found the incidence of BP to be significantly higher for people in their ninth or tenth decade than for people of other ages (58, 59). Thus, aging has been recognized as a significant risk factor for BP onset. Several studies investigated the prevalence of anti-BP180 autoantibodies in the elderly (40, 45–49, 60). Interestingly, Hachisuka et al. showed no preclinical anti-BP180 autoantibodies in 32 normal elderly individuals (46). In addition, large retrospective studies using unaffected subjects or a general population in Japan did not find a significant difference between age and the prevalence of anti-BP180 autoantibodies (39, 44).

Pruritus is a common problem among elderly people (61), and pruritus may develop in the early stage of BP (2, 62–64). Several studies reported the prevalence of preclinical anti-BP180 autoantibodies in elderly patients with pruritic disorders (40, 47–49). Hofmann et al. showed that three (12.0%) of 25 elderly patients with pruritic disorders had anti-BP180 autoantibodies, and the mean titer ± SD of these positive sera was 18.1 ± 4.6 (47). However, DIF, ssIIF, and immunoblotting using cultured keratinocyte extracts were negative among all elderly patients with pruritic disorders. Feliciani et al. investigated preclinical anti-BP180 autoantibodies using ELISAs with the mid-portion (aa490–812) or C-terminus (aa1048–1465) of BP180 in elderly patients with pruritic disorders (48). Both BP180 mid-portion and C-terminus ELISAs detected preclinical BP autoantibodies in two (13.3%) of 15 elderly patients with pruritic disorders, whereas one (4.0%) of 25 elderly patients with immediate-type allergy had preclinical anti-BP180 autoantibodies. Fania et al. showed a higher prevalence of preclinical anti-BP180 autoantibodies (23.8%) in elderly patients with pruritic disorders than that in elderly patients with allergic diseases (4.2%) by BP180 N-terminus and C-terminus ELISAs (49). In contrast, van Beek et al. found no significant difference in the prevalence of anti-BP180 autoantibodies among elderly patients with chronic disorders or non-inflammatory skin diseases and blood donors using a BP180 NC16A ELISAs (40). These results are conflicting, and further studies are needed.

### Collagen disease

Several case reports showed that collagen diseases such as systemic lupus erythematosus, dermatomyositis, rheumatoid arthritis, or systemic sclerosis can co-exist with BP (65–73). Although it remains unclear whether collagen diseases are a risk factor for BP development, two studies explored preclinical anti-BP180 autoantibodies in a collagen disease population while evaluating the diagnostic performance of BP180 NC16A ELISAs (6, 16). These studies showed the prevalence of anti-BP180 autoantibodies to be 1.1–8.0% in collagen disease patients, which is comparable with the 1.5–2.0% in healthy controls.

### Neurological disease

Previous epidemiological studies suggest that neurological diseases are a risk factor for BP development (74–76). A Finnish epidemiological study indicated that multiple sclerosis (MS) and Alzheimer’s disease increase the risk of BP development (77, 78). As neurological diseases are associated with BP, several studies examined the prevalence of preclinical anti-BP180 autoantibodies in patients with neurological disorders (42, 43, 50, 51).

Messingham et al. investigated the prevalence of BP autoantibodies in patients with Parkinson’s disease and dementia using a BP180 NC16A ELISA and immunoblotting using the BP180 ectodomain or intracellular domain (50). BP180 NC16A ELISA detected BP autoantibodies in 3.8% of the dementia patients but not in any of the Parkinson’s disease patients nor in the control subjects. Immunoblotting using the BP180 ectodomain found BP autoantibodies in 29.2% of the Parkinson’s disease patients, whereas immunoblotting using the BP180 intracellular domain detected BP autoantibodies in 19.2% of the dementia patients. Tuusa et al. indicated that BP autoantibodies were detected in 11.8% of MS sera with the full-length BP180 ELISA, while BP autoantibodies were detected in 53.8% of MS sera with immunoblotting using the full-length BP180, suggesting that BP autoantibodies from MS sera preferentially react to the denatured form of BP180 but not to its native form (43). A conflicting result was reported by Recke et al. (42). Although BIOCHIP, BP180 NC16A ELISA, BP230 ELISA, and immunoblotting with the extracellular matrix of cultured human keratinocytes were used to detect anti-BMZ antibodies in MS and Parkinson’s disease patients, MS and Parkinson’s disease showed no significant increased prevalence of BP autoantibodies (42). Wang et al. recently reported that anti-BP180 NC16A autoantibodies were found in 14 (14.0%) of 100 patients with stroke, and the mean titer ± SD was 19.2 ± 6.1 (52). Among these patients, 12 (85.7%) of the 14 positive sera were also positive by immunoblotting using BP180 recombinant proteins. The prevalence of BP autoantibodies detected by BP180 NC16A ELISA was found to be 18.0% in Alzheimer’s disease patients (51). Interestingly, subsequent analysis found the severity of dementia to correlate significantly with the titration of anti-BP180 NC16A autoantibodies in Alzheimer’s disease (51). In addition, Wang et al. reported that 20 of 48 (47.9%) patients with Alzheimer’s disease had anti-BP180 autoantibodies detected by BP180 NC16A ELISA, and the mean titer ± SD was 22.9 ± 20.9 (53). Among these patients, 9 (64.3%) of the 14 positive sera were also positive by immunoblotting using BP180 recombinant proteins.

### Diabetes mellitus

BP may initially mimic other pruritic dermatoses, and it is known that DM is often associated with pruritic dermatoses (79). Therefore, the potential association between DM and the early phase of BP has been discussed. To address this issue, Jedlickova tested the seropositivity of anti-BMZ antibodies by IIF and ELISAs using recombinant BP180, BP230, and Laminin 332 (54). There were no significant differences in the seropositivity of anti-BMZ autoantibodies among non-diabetic patients or diabetic patients with or without pruritus; however, the prevalence of anti-BMZ autoantibodies in all groups detected by IIF was relatively high (12.2%). Recently dipeptidyl peptidase-IV inhibitors (DPP4i), medications for type 2 diabetes treatment, have attracted interest as a causative drug of BP (80–86). Izumi et al. investigated the prevalence of anti-BMZ autoantibodies among 275 DM patients with or without DPP4i administration (55). BP180 NC16A ELISA, BP230 ELISA, and full-length BP180 ELISA detected BP autoantibodies in 1.8%, 2.2%, and 10.9% of 221 DM patients with DPP4i treatment, respectively, whereas these assays detected BP autoantibodies in 0%, 7.4%, and 5.6% of 54 DM patients without DPP4i treatment, respectively. Among these patients, 54.2% of full-length BP180 ELISA-positive sera were also positive by ssIIF. Although there were significant differences in the prevalence of anti-full-length BP180 IgG between DM cases with and without DPP4i treatment in this study, the anti-full-length BP180 autoantibody-positive cases are likely to have been significantly older than the anti-full-length BP180 IgG-negative cases in the DM cases with DPP4i.

### Other skin diseases

For the validation of anti-BP180 autoantibody detection, several studies performed the detection of anti-BP180 autoantibodies in patients with various skin diseases as a negative control for the detection system (16, 76). van Beek et al. reported that 7.2% of patients with non-inflammatory skin diseases, including leg ulcers, basal cell carcinoma, and squamous cell carcinoma, had anti-BP180 autoantibodies detected by BIOCHIP, although the details were not mentioned (22). For investigating the prevalence of anti-BP180 autoantibodies in elderly patients with pruritic disorders, elderly patients with allergic disorders or with non-inflammatory skin diseases were used as a control group (48, 49). In these studies, positive rates of anti-BP180 autoantibodies were 0.0*–*4.2% in elderly patients with allergic disorders detected by ELISAs using BP180 aa490*–*812 and aa1048*–*1465 (48, 49), and 1.3*–*3.2% in elderly patients with non-inflammatory skin diseases (40).

In addition, Powell et al. examined whether BP180 NC16A ELISA was able to differentiate pemphigoid gestationis, which is a blistering autoimmune disease mediated by anti-BP180 autoantibodies during pregnancy, from polymorphic urticarial papules and plaques of pregnancy (PUPPP) (37). In this study, 4.9% of the PUPPP patients showed seropositivity with BP180 NC16A ELISA.

## Prodromal BP

The diagnosis of BP is usually made by the combination of clinical, histopathological, and immunological findings described above. Typical tense blisters are an essential clue in diagnosing BP, and it is challenging to diagnose BP in cases without blisters. A prospective nationwide cohort study found that BP was diagnosed an average of 6.1 months after onset of the first symptoms (87). Cohort studies showed that 17–20% of BP presented with no apparent blisters at the time of BP diagnosis (2, 87). There is the concept of prodromal BP (62, 88–90). Retrospective studies showed that 31.8% and 36.8% of BP patients had non-bullous lesions before the appearance of typical blisters (90, 91). The duration before blister development ranged from 2 weeks to 19 years: That duration was up to 12 months for 82% of the patients in the Sun et al. study, and the mean duration before blister appearance was 15.9 months in the Zhang et al. study (90, 91). This non-bullous stage of BP may lead to a delayed diagnosis.

Also, a subtype called non-bullous pemphigoid has been reported (63, 92–94). In a systematic review of non-bullous pemphigoid described by Lamberts et al., the most common clinical presentations of patients with non-bullous pemphigoid were erythematous, urticarial plaques and papules/nodules, and 9.8% of the non-bullous pemphigoid patients developed bullae during the reported follow-up (63). In addition, an IgM autoantibody-mediated pemphigoid disease called IgM pemphigoid has been reported (95–97), and IgM autoantibodies targeting BP180 might play a pathogenic role in IgM pemphigoid (95, 96). It is debatable whether non-bullous pemphigoid and IgM pemphigoid are just prodromes of BP or are distinct pemphigoid variants.

The detection of anti-BP180 autoantibodies might be an indicator for subsequent BP development. Wang et al. retrospectively analyzed medical records from 2005 to 2015 from a single center and found 208 BP autoantibody-positive patients with BP180 and/or BP230 ELISA but not with DIF (98). Dermatitis was the most common diagnosis among preclinical BP autoantibody-positive patients; of note, four patients had positive DIF results upon repeating the tests, and a diagnosis of BP was made during follow-up (98). Raneses et al. showed that 4 of 18 BP patients had positive ani-BP180 or anti-BP230 autoantibodies 79 months (range: 0.1 to 217 months) before the onset of BP symptoms (9).

## Concluding remarks

Certain patients with various medical conditions such as aging with pruritus, neurological diseases, and diabetes mellitus with the administration of DPP4i can have anti-BP180 autoantibodies without BP development. However, it remains controversial whether the existence of anti-BP180 autoantibodies is a predictive factor for BP development. Further studies on preclinical BP autoantibodies promise to reveal whether the presence of anti-BP180 autoantibodies is a predictive marker for BP development and to identify the critical factors contributing to BP onset in the presence of BP autoantibodies.

## Author contributions

YM, KI, and SM drafted the paper. HU supervised the writing of the manuscript. All authors contributed to the article and approved the submitted version.

## Conflict of interest

The authors declare that the research was conducted in the absence of any commercial or financial relationships that could be construed as a potential conflict of interest.

## Publisher’s Note

All claims expressed in this article are solely those of the authors and do not necessarily represent those of their affiliated organizations, or those of the publisher, the editors and the reviewers. Any product that may be evaluated in this article, or claim that may be made by its manufacturer, is not guaranteed or endorsed by the publisher.

## Abbreviations

BP, bullous pemphigoid; BMZ, basement membrane zone; NC16A, the 16th non-collagenous A domain; ELISA, enzyme-linked immunosorbent assay; DIF, direct immunofluorescence analysis; IIF, indirect immunofluorescence analysis; ssIIF, 1M NaCl-split skin indirect immunofluorescence analysis; SD, standard deviation; MS, multiple sclerosis; DM, diabetes mellitus; DPP4i, dipeptidyl peptidase-IV inhibitor; DPP4i-BP, dipeptidyl peptidase-IV inhibitor-associated bullous pemphigoid; PUPPP, polymorphic urticarial papules and plaques of pregnancy.
